# Extracorporeal Photopheresis: Tolerogenic or Immunogenic Cell Death? Beyond Current Dogma

**DOI:** 10.3389/fimmu.2015.00349

**Published:** 2015-07-07

**Authors:** Dalil Hannani

**Affiliations:** ^1^PDC*line Pharma SAS, Grenoble, France

**Keywords:** extracorporeal photopheresis, GvHD, CTCL, transplantation, cancer, T cell vaccination, anti-clonotypic response, immunogenic cell death

Extracorporeal photopheresis (ECP) is an autologous cell therapy that is widely used for the treatment of T cell-mediated diseases. ECP has been FDA-approved for the treatment of cutaneous T cell lymphoma (CTCL) and has shown potent clinical benefits in various other (non-cancer) T cell-mediated diseases, such as graft versus host disease (GvHD), allograft rejection, as well as in autoimmune disorders, such as rheumatoid arthritis, psoriasis, systemic sclerosis, type 1 diabetes, and Crohn’s disease (1–3). The ECP treatment consists in the irradiation by UV-A in presence of a photosensitizer agent (8-methoxypsoralen) of PBMCs collected by apheresis (4). This will lead to an irreversible DNA crosslink by the psoralen, culminating by the apoptosis of virtually all the treated cells (5, 6). Then, the treated cells are re-infused to the patient. This repeated process leads to the improvement in patients’ clinical status, allowing the decrease or the disappearance of tumoral T cells in CTCL, or a decrease or a total disruption of immunosuppressive drugs, thus avoiding steroid-related side effects in GvHD (7). ECP has also shown benefits in cortico-refractory patients (8). Conversely to immunosuppressive treatments, ECP seems to selectively target allo- and auto-reactive T cells in GvHD and autoimmune diseases, respectively (called pathogenic T cells hereafter), without inducing systemic immunosuppression (9). Today, even if ECP has created real hopes for the treatment of these pathologies, its implementation is quite limited due to a relative empiric utilization due to the absence of prospective randomized clinical trials and a lack in the understanding of its mechanism of action (MoA).

For instance, ECP is thought to act through the induction of immune tolerance in GvHD. Indeed, Gatza et al. have described that the injection of ECP-treated splenocytes from mice developing GvHD (i.e., containing allogeneic T cells) triggers IL-10-producing regulatory T cells (Tregs) able to reverse experimental GvHD (10). However, authors did not assess whether or not ECP-induced Tregs were alloantigen specific (i.e., that ECP does not induces a systemic tolerance in this setting) in order to fully recapitulate the clinical situation observed in humans.

The infusion of apoptotic cells has previously been described as promoting tolerance. Notably, the infusion of γ-irradiated apoptotic splenocytes, concomitantly with bone marrow cells, triggers the generation of TGF-β-dependent Tregs, which in turn favors the bone marrow cells’ engraftment as well as protects from GvHD occurrence (11). A similar approach has recently been evaluated in a phase I/IIa clinical trial as prophylaxis for GvHD, where donor apoptotic cells have been injected to recipient 1 day before bone marrow transplantation (BMT), and has shown encouraging results (12). In line with this, some studies proposed to use ECP-treated autologous cells as a prophylactic treatment of GvHD (13). This therapeutic setting prevents or at least diminishes the occurrence of acute GvHD by inducing Tregs, in a host IL-10-production-dependent manner in mice. In both of these settings, prior massive infusion of apoptotic cells might induce systemic immune tolerance, which in turn diminishes or prevents acute GvHD development following BMT. In an *in vitro* model, Di Renzo et al. have shown that monocyte-derived dendritic cells (DCs) from GvHD patients secreted an increased amount of IL-10 when stimulated by LPS in presence of autologous ECP-treated T cells (14).

Altogether these data indicate that ECP might be able to induce, at least in part, immune tolerance. However, the generation of Tregs as a unique mechanism neither explains how ECP selectively targets pathogenic T cells without inducing a systemic immunosuppression (9) nor how it works in CTCL. Indeed, the hypothesis that has been made concerning its MoA in CTCL is rather the elicitation of an anti-tumor response directed toward tumoral T cells (9). How ECP could trigger both an anti-tumor immune response and immune tolerance remains an open question.

The pathologies treated by ECP are heterogeneous; however, they are all mediated by a (oligo)-clonal T cell population (tumoral T cell clones in CTCL, allo- or auto-reactive oligoclonal T cells in GvHD and autoimmune diseases). Thus, these T cells share unique or a few T cell receptors (TcR) representing pathogenic T cell-specific antigens that can be subsequently targeted by ECP-induced immune responses.

Importantly, the presence of this pathogenic T cell population within the treated cells is critical for the ECP efficacy (15). This observation has also been made recently in an animal model in which ECP was efficient only when pathogenic, and not naive T cells, were treated (16). Interestingly, these observations are in line with the seminal work pioneered by Irun Cohen that has developed the T cell vaccination (TCV) concept, showing that the injection of altered activated pathogenic T cells results in the systemic control of untreated pathogenic T cells by triggering anti-clonotypic cytotoxic CD8 T cells. (17–19). Of note, clinical trial have been performed, using TCV in multiple sclerosis (MS), and have shown very encouraging results (20). Zhang and colleagues have been the firsts testing this concept in humans (21). In this study, MBP-reactive T cells have been isolated and amplified *ex vivo* from MS patients. Then, amplified activated pathogenic T cells have been irradiated and then infused back to the patient. This treatment has led to clinical responses, illustrated by the disappearance of pathogenic T cells (i.e., untreated MBP-reactive T cells). This clinical response is due to the generation of anti-clonotypic CD8 T cells, which are able to eliminate pathogenic T cells in a cytotoxic-dependent manner (21). Thus, altogether, these critical data underlie the necessity of providing dying pathogenic T cells (containing specific antigens) in order to obtain a therapeutic response, evoking an anti-(oligo)clonotypic immune response triggered by the repeated re-infusion of treated pathogenic T cells.

Until recently, apoptosis has been described as a silent/tolerogenic process, where dying cells either die «silently» or actively secrete – and/or induce the production of – anti-inflammatory cytokines, such as IL-10 and TGF-β (22). During the last decade, Zitvogel’s group has published a seminal work describing that in particular conditions, tumor cell death could be an immunogenic process, able to elicit an immune response directed toward this population (23, 24). At least four events are mandatory for undergoing an immunogenic cell death (ICD) as follows: (1) the membrane exposure of calreticulin (CRT) following a pre-mortem endoplasmic reticulum (ER) stress response. This favor the phagocytosis of dying cells (25); (2) the release of a nuclear protein that acts as an alarmin in the extracellular environment, high-mobility group box (HMGB)-1 (26); (3) the release of ATP that favors the production of IL-1β (27) as well as the attraction and differentiation of antigen presenting cells (APCs) (28); and (4) the activation of autophagy machinery that is critical for ATP release (29). ICD has been described in the context of chemotherapy-induced tumor cell death, and has been recently evidenced following photodynamic therapy (30). However, to date, whether ECP induces ICD is still unknown.

Extracorporeal photopheresis-induced ICD would support the anti-clonotypic response hypothesis in CTCL. Indeed, the re-infusion of tumoral T cells undergoing ICD back to the patient would facilitate DC-mediated phagocytosis as well as DC maturation. Of note, Yakut et al. have shown that ECP actually promotes IL-1β production by ECP-treated DCs (31). Since IL-1β is a key cytokine involved in ICD-induced anti-tumor responses (23, 24, 27), IL-1β-producing DCs would be, in turn, able to initiate an anti-tumor immune response directed toward living cancer cells. Importantly, ECP-induced ICD could also support this hypothesis in GvHD (and solid organ transplantation and autoimmunity disorders) as well. Indeed, in these pathologies, oligoclonal pathogenic T cells may represent an important proportion of circulating T cells – therefore, an important proportion among treated cells – and are in an activated state (because of undergoing allo- or auto-immunity). Interestingly, it has been shown that activated T cells die more rapidly than resting T cells following ECP treatment (32). It means that during the first hours following re-infusion of treated cells, only pathogenic T cells undergo (immunogenic?) cell death. In these conditions, they become the unique source of antigens. Thus, this window of time allows the preferential phagocytosis of dying pathogenic T cells by APCs, and subsequent antigen processing and presentation to the immune system. Of note, Johansson and colleagues have shown that in presence of activated, but not resting, apoptotic T cells, autologous DCs acquired a mature phenotype and produce pro-inflammatory cytokines (33). Importantly, DCs exposed to allogeneic, activated apoptotic T cells induce the proliferation and IFNγ production by autologous T cells. In this setting, pathogenic activated T cell TcR-derived peptides could be presented to the immune system, leading to the elicitation of an anti-(oligo)clonotypic immune response, targeting the pathogenic (oligo)clonal T cell population (Figure 1). This scenario would explain why the presence of pathogenic T cells is critical for reaching therapeutic success, as well as, how ECP induces a specific control of alloreactive T cells responsible for GvHD and solid organ rejection, or autoimmune T cells involved in autoimmune disorders, without inducing generalized immunosuppression (i.e., by eradicating specifically the pathogenic T cells). Ayyildiz et al. have reported that the serum TNF-α level decrease 1 day after ECP treatment in chronic GvHD (34). Interestingly, during the first ECP treatments, the serum TNF-α level fluctuates and it is found as high as baseline prior to the second ECP treatment. It is likely that ECP first induces a transient immune tolerance due to the infusion of large quantity of apoptotic cells, as described in other settings (11, 13). However, following several ECP sessions, the serum TNF-α level tends to stably decrease in responding patients (34). It is conceivable that ECP-induced transient immune tolerance could be paralleled and/or followed by the generation of anti-clonotypic responses, which would indirectly trigger a steady TNF-α decrease by eliminating pathogenic T cells. Indeed, ECP-induced ICD of pathogenic T cells could reconcile the apparently contradictory MoAs proposed so far (triggering immunity in CTCL and immune tolerance in GvHD). Understanding ECPs MoA will help considerably in rationalizing treatment schedules and processes as well as its application field. Finally, it is a critical step toward identifying a predictive biomarker of efficacy for improving the patients’ response rates and for proposing synergizing combinatory therapy for rescuing unresponsive patients.

**Figure 1:**
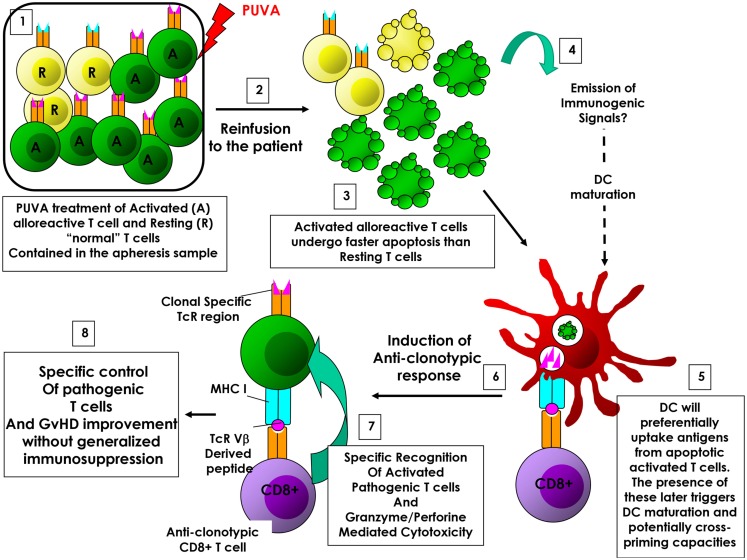
**ECP-induced anti-clonotypic response in GvHD**. (1) (Oligo)clonal activated alloreactive T cells are enriched compared to «normal» resting T cells among treated cells. (2) Cells are re-infused back to the patient. (3) Activated T cells undergo apoptosis faster than resting T cells. (4) Emission of immunogenic signals. (5) During this window of time, dying activated T cells will be preferentially phagocytized by dendritic cells (DCs), representing the main source of antigen. (6) DCs will then process and present alloreactive associated T cells antigens (i.e., TcR-derived peptides), allowing the elicitation of an anti-clonotypic response. (7) Anti-clonotypic T cells will then specifically recognize and eradicate alloreactive T cells. (8) The eradication of alloreactive T cells will lead to the improvement of GvHD without inducing systemic immunosuppression.

This short opinion article provides an original point of view in this field and proposes a MoA in which ECP induces an immunogenic, rather than a tolerogenic, cell death. This scenario is the only one describing a unique MoA able to explain the efficacy of ECP in such different pathologies, and therefore, strongly deserves to be fully investigated.

## Conflict of Interest Statement

The author declares that the research was conducted in the absence of any commercial or financial relationships that could be construed as a potential conflict of interest.
